# Motivations, delivery, perceived benefits, and barriers to delivery of the *parkrun* practice initiative in general practices across the UK: a national cross-sectional online survey of healthcare professionals and event organisers

**DOI:** 10.1186/s12875-025-02827-9

**Published:** 2025-04-29

**Authors:** Callum J. Leese, Ross Clarke, Robert H. Mann, Rosina Cross, Hussain Al-Zubaidi, Blair H. Smith, Emma J. Cockcroft

**Affiliations:** 1 Department of Population Health and Genomics, University of Dundee, Ninewells Hospital, James Arrott Drive, Dundee, UK; 2 University of Exeter Medical School, Exeter, UK; 3 Department of Public Health and Sport Sciences, University of Exeter Medical School, Exeter, UK; 4 Department of Health and Community Sciences, University of Exeter Medical School, Exeter, UK; 5 Physical Activity and Lifestyle Champion, Royal College of General Practitioners, London, UK

**Keywords:** General practice, Physical activity, Exercise, Engagement, Social prescribing, Community

## Abstract

**Background:**

Physical activity offers significant health benefits, yet many people in the United Kingdom do not meet recommended guidelines. Primary care plays a crucial role in physical activity promotion, but barriers can hinder implementation. The *parkrun* practice initiative, launched in 2018, aims to address these barriers by linking general practices with local *parkrun* events.

**Aim:**

This study aimed to evaluate the *parkrun* practice initiative from the perspective of staff at general practices and parkrun event organisers, exploring the motivations for joining, the variety of ways in which the initiative was delivered, perceived benefits on patients and staff, and barriers to implementation.

**Methods:**

A cross-sectional online survey was distributed via email to 1,852 registered *parkrun* practices and 800 ‘linked’ *parkrun* event organisers. Descriptive statistics were used to present quantitative data. Content analysis was used to analyse qualitative data.

**Results:**

Responses from 416 staff at *parkrun* practices (22% of registered practices) and 439 event organisers (55% of all events organisers) were included in the analysis. Attendance of staff at the local *parkrun* and sharing of information with patients were the main means of initiative implementation. Our findings highlight the perceived benefits of the initiative on staff and patient health and wellbeing, *parkrun* practice staff morale, and community engagement. A discrepancy is noted between what is being done by practices and what is being perceived by event organisers. Major barriers to implementation included: a lack of time; a lack of engagement of practice staff; the COVID-19 pandemic; and access to the nearest *parkrun*.

**Conclusion:**

To address the barriers in implementing the *parkrun* practice initiative in primary care, our findings indicate that future initiatives should look to include: (1) clear and ongoing communication to ensure widespread engagement of patients, staff and event organisers; (2) ease of implementation (minimising time demands); and (3) adequate resource allocation to facilitate implementation (e.g., financial, educational, personnel). Further research is required to increase understanding of the impact on patient outcomes, staff morale, and the behavioural mechanisms influencing initiative implementation.

**Supplementary Information:**

The online version contains supplementary material available at 10.1186/s12875-025-02827-9.

## Introduction

Evidence shows that physical activity (PA) is associated with physical, psychological, and social health benefits [1–3], including reduced risk of all cause and cause specific mortality [4]. Due to the widely reported benefits of PA, it is upheld as an essential tool for both the prevention and management of Non-Communicable Diseases (NCDs). NCDs are accountable for approximately 41 million deaths globally per year, constituting ~ 74% of global deaths [5]. Notably, four disease categories—cardiovascular (~ 17.9 million annually), cancer (~ 9.3 million annually), chronic respiratory disease (~ 4.1 million annually), and diabetes (~ 2.0 million annually, inclusive of diabetes-induced kidney disease deaths)—contribute to over 80% of premature fatalities from NCDs [5]. In 2019, the Chief Medical Officers (CMOs) for the United Kingdom (UK) introduced updated PA guidelines, recommending that adults should aim to accumulate at least 150 min of moderate intensity aerobic exercise per week, including at least two weekly sessions aimed at muscle strengthening and balance [6]. However, approximately one-third of adults in the UK do not meet these PA guidelines [7].

Primary care is a key point of influence for addressing physical inactivity as it is the first point of contact individuals have with the health system– providing greater exposure to the whole population than any other health professional [8, 9]. Primary care professionals also regularly see those in need of PA advice and are viewed by the public as a trusted source of information [8, 9]. Growing evidence supports the effectiveness of PA promotion delivered in primary care at increasing PA in patients [10–13]. PA promotion in primary care has also been shown to be one of the most cost-effective approaches to promote PA at a population level [14]. Despite this, research has repeatedly evidenced a lack of PA promotion across primary care in the UK [15, 16]. Previous work has explored the reasons for this [17, 18], including the identification of four main barriers to the delivery of PA promotion faced by staff in primary care. These relate to a lack of: (1) time to promote PA; (2) resource/support to deliver promotion; (3) knowledge of how to deliver PA promotion, particularly in disease specific population; and (4) financial reimbursement.

Community-based interventions exist that promote PA participation at the individual and population level. One example is *parkrun*– a charity that delivers free, weekly, and timed five-kilometre walks or runs for all ages in parks and green-spaces across the UK (and in 21 other countries around the world). The events are non-competitive and focus on participation and inclusion. To help address low levels of PA promotion in primary care in the UK, the *parkrun* practice initiative was launched in 2018, in affiliation with Sport England. This collaboration between the Royal College of General Practitioners (RCGP) and *parkrun* UK is a social prescribing project that encourages practices of all sizes to link with their local *parkrun* events to improve physical activity levels in both patients and staff.

The *parkrun* practice website hosts information related to the *parkrun* practice initiative, including a toolkit with ideas for activities that practices can undertake in order to encourage patients and staff to become more active [19]. These suggested activities include (but are not limited to): sharing written information with staff and patients, presence of a noticeboard, delivering talks and presentations, advertising in waiting rooms and regularly talking to colleagues and patients about parkrun and the benefits of participating. Event organisers (EOs) are also provided with a bespoke toolkit, enabling them to support general practices in the delivery of the initiative. A practice is registered as a *parkrun* practice following approval from an RCGP representative in response to submission of appropriate paperwork. As documented in previous research [20], the benefits of *parkrun practices* are wide ranging, and include improved mental and physical health and an enhanced sense of community. At the time of writing approximately 1,900 practices are signed up to the initiative, representing ~ 31% of all UK practices.

An early evaluation of the initiative was conducted in 2019 and published in 2020 [21]. This mixed-methods study of 306 *parkrun* practices identified several key motivators for participation (e.g., improving patient and staff health and wellbeing and facilitating community engagement) and several key barriers (e.g., lack of time and engagement). Since then, the number of *parkrun* practices (and *parkrun* events) has continued to grow (i.e., the number of parkrun practices more than doubled from 780 to over 1,900). Given the proliferation in numbers, passage of time, and increasing burden on primary care in the UK– due to the COVID-19 pandemic, funding shortfalls, and staffing shortages across practices– there is a need to re-evaluate the *parkrun* practice initiative. Furthermore, given its widespread adoption in the UK (31% of all GP practices), it can also provide lessons to help inform other PA promotion initiatives in healthcare settings (e.g., the Active Practice Charter [22]).

This study aimed to evaluate the *parkrun* practice initiative from the perspective of both general practices and parkrun event organisers, exploring the motivations for joining, means of delivery, the perceived benefits on patient and staff physical activity, and barriers to implementation.

## Methods

### Survey design and distribution

Two surveys were developed for this study– one aimed at practice staff, and one aimed at *parkrun* EOs. These surveys were developed by an advisory panel of experts including academics (*n* = 3), General Practitioners (GPs) with a special interest in PA (*n* = 2), and *parkrun* representatives (*n* = 2). The surveys included a mixture of Likert scale, closed, and free-text response questions (see Supplementary File 1). Question development was informed by the toolkit provided by the RCGP and *parkrun* to practices to support them setting up and delivering the initiative [23]. Once developed, an online version of each survey was created using the Jisc Online Survey (JOS) tool.

Specifically, the survey for practice staff was designed to assess: (1) practice population demographics; (2) motivations for practices joining the initiative; (3) their means of implementing the initiative; (4) the perceived benefits of the *parkrun* practice initiative on patients and staff, and (5) barriers to implementation. The EO survey was designed to assess: (1) demographics (2), perceived benefits of the initiative on the *parkrun* event (3), the EO perceptions of how the initiative is delivered by practices and (4) barriers to implementation.

The *parkrun* practice staff survey was distributed via email by a RCGP Senior Project Manager to the contacts registered in the RCGP database for all practices that had achieved *parkrun* practice status. This represented 1,852 practices at the date of distribution (5th April 2024).

The EOs survey was sent to all *parkrun* UK events via email by the communications team at *parkrun* Global (*n* = 800). The surveys were also shared via RCGP and *parkrun* social media channels and newsletters.

One representative from each practice or *parkrun* event was asked to complete the survey. Reminder emails were sent by the same RCGP Senior Project Manager and the *parkrun* team at both two (19th April 2024) and four weeks (3rd May 2024) following initial contact, with the surveys closing after six weeks (17th May 2024). Informed consent was obtained prior to participation in the survey. Participation was voluntary and unpaid, with completion of the survey being possible via desktop or mobile devices. All participants were invited to be included into a prize draw (prizes were 1 pair of running shoes, 2 pairs of headphones and 3 *parkrun* t-shirts) on completion of the survey.

Ethics for non-clinical research was sought and approved by the University of Dundee’s Research Ethics Committee (UOD-SMED-SLS-Staff-2023-23-98).

### Participants

Only *parkrun* EOs or employed staff (both clinical and non-clinical) of accredited *parkrun* practices in the UK were invited to participate. Responses from practices that did not have *parkrun* practice status were ineligible, with any responses from these practices deleted. In the event of multiple responses from the same individual, the first response was retained and the remainder removed from the final analysis. Similarly in the event of multiple responses from different individuals at the same *parkrun* event or practice, the first response was retained and the remainder removed from the final analysis.

### Data analysis

Data were downloaded and cleaned in Microsoft Excel (Version 2402) before descriptive statistics were used to present demographics data, closed question responses, and Likert scale question responses.

Survey responses for the free-text (open-ended) questions were analysed using a content analysis approach, as described by Hsieh and Shannon [24]. A predominantly inductive and semantic style of content analysis was employed. The analysis involved the following five stages: (1) all free text responses were read by two authors (CL, RC) to ensure familiarisation with the data; (2) data were divided into responses to three areas (motivation for joining, barriers to implementation and perceived benefits) identified at the survey creation and directed by the research question (CL); (3) a coding framework was developed for each subordinate area by two authors (CL and RC) in collaboration; (4) the free text was analysed line by line and coded into sub-categories by one author (RC or CL) with a 10% check by the other author; (5) generated codes were categorised into themes according to similarities and differences by one author (RC) and discussed and agreed upon by another (CL); (6) a frequency analysis of generated themes was conducted to explore whether certain barriers were experienced more frequently than others. At each stage of the data analysis two authors (RC and CL) met to discuss interpretations and congruence of these in relation to the themes being generated. Any unresolved congruences were discussed with additional authors (EC, RM, and RCr) during regular meetings at key stages.

## Results

### Sample characteristics

At the time of survey distribution, there were 1,852 registered *parkrun* practices and 800 *parkrun* events in the UK. There were 855 responses to the survey, after 28 duplicates were removed. Fifteen of these were removed due to responses being from the same practice, and 13 removed due to responses being from the same individual.

### Demographics

439 EOs were included in the analysis, representing 55% of total *parkrun* events in the UK. Of these respondents, 207 reported that their event was linked with one or more GP practice. Of the *parkrun* events who stated that they were knowingly linked to a practice, the majority were linked with only one practice (*n* = 100, 48%), whilst 25% were linked with two practices (*n* = 52), 10% with three practices (*n* = 22), and 16% with four or more (*n* = 33).

The remaining 232 EOs that responded reported that their event was not (as far as they were aware) linked with a local GP practice. Sixty-five of these responders (28%) reported having tried unsuccessfully to link with a GP practice.

Responses from 416 practices were included in the final analysis. This represents 22.5% of all *parkrun* practices at time of dissemination of survey. Practice and practice staff demographics are shown in Table 1.

**Table 1:** Practice and healthcare responder characteristics

Practice Characteristics	*n* (%)
**Practice List Size**	
< 4,000	10 (2.4%)
4,000–7,999	74 (17.8%)
8,000–11,999	122 (29.3%)
12,000–15,999	90 (21.6%)
16,000–20,000	51 (12.3%)
> 20,000	169 (16.6%)
**Number of** ***parkruns*** **linked with practice**	
1	344 (82.7%)
2	55 (13.2%)
3	6 (1.4%)
4 or more	11 (2.6%)
**One or more parkruns within catchment area**	
Yes	301 (72.4%)
No	115 (27.6%)
**Length of affiliation**	
< 1 year	69 (16.6%)
1–2 years	67 (16.1%)
2–3 years	114 (27.4%)
4 or more years	166 (39.9%)
**Establishing Links with Parkrun**	
Parkrun event made first contact	46 (11.1%)
Practice made first contact	349 (83.9%)
Other	21 (5.0%)
**Healthcare Responder Characteristics**	**n (%)**
**Responder role**	
General Practitioner	266 (63.9%)
Nurse	25 (6.0%)
Practice Manager	66 (15.9%)
Administration team	24 (5.8%)
Doctor in training	3 (0.7%)
Physiotherapist	3 (0.7%)
Other	29 (7.0%)
**Prior Parkrun Participation**	
Yes	334 (80.3%)
No	77 (18.5%)
Can’t remember	5 (1.2%)

### Motivation for joining the initiative

Table 2 summarises the reasons identified by practice staff for registering their practice as a *parkrun* practice.

**Table 2:** A summary of the qualitative content analysis for the survey assessing the motivation of practice staff for joining the parkrun practice initiative

Theme	Codes included	Percentage of responses (*n* = 416) Raw value in brackets	Illustrative quotes:
Health and Wellbeing	Patient Health/Wellbeing	65.4% (272)	*“I am a GP and a runner*,* and know the benefits of running on mental and physical health*,* and am keen to promote physical activity to my patients. Parkrun is very inclusive and would hugely benefit so many people if they got involved.” (HCW68)*
	Staff Health/Wellbeing	43.0% (179)	*“Awareness of*,* and desire to promote / harness*,* the physical*,* social and mental health benefits of running and being involved with the parkrun community for our patients and staff.” (HCW62)*
	Community Engagement	10.8% (45)	*“As a GP I have seen the benefits of parkrun and feels it should benefit my patients either as preventive measure or as a treatment. Parkrun is also like a community which help people to socialize.” (HCW83)*
	Staff Morale	6.0 (25)	*“Physical and mental health promotion*,* team building and meeting with people.” (HCW149)*
	Alignment with Practice Values	4.1% (13)	*“Promoting healthy lifestyles is a priority for the practice.” (HCW174)*
Direct Engagement with Parkrun	Prior Parkrun Participation	38.5% (160)	*“A number of staff already took part in parkrun and also have an interest in lifestyle medicine so it was a natural progression.” (HCW28)*
	Event Organiser Engagement	3.4% (14)	*“Speaking with the local parkrun event team.” (HCW20)*
	Parkrun Publicity	1.4% (6)	*“A flyer was sent out to us by the parkrun organiser and we thought it was a great initiative that would benefit our patients.” (HCW301)*
Promotion from other sources	RCGP Advocacy	5.5% (23)	*“RCGP Active Practice Charter.” (HCW24)* *“RCGP initiative seen by partner who has been a participant.” (HCW423)*
	Other Link Initiatives	3.1% (13)	*“It was advised by local ICB.” (HCW406)*
Abbreviations: RCGP, Royal College of General Practitioners; ICB, Integrated Care Board; ANP, Advanced Nurse Practitioner			

Alongside the motivation of improving patient (65.4%, n = 272) and staff (43.0%, n = 179) wellbeing, and personal advocacy of already being an active *parkrun* participation (38.5%, n = 160), respondents highlighted several other motivations. Community engagement (10.8%, n = 45) reflects a desire to meet patients and fellow colleagues outside the work environment, acknowledging the power of socialisation, as well as benefits on staff morale which was also independently expressed as a motivation (6.0%, n = 25):

*“[…] very sociable and good to meet everyone away from work with a purpose– we all look forward to it” (HCW431)*.

Respondents also cited the role of the RCGP advocacy (5.5%, n = 23). The RCGP have a wide distribution network and are a trusted organisation. This platform was used to deliver the initiative and advertise the initiative:

*“I organised it [active practice charter] having seen a talk at RCGP conference years ago” (HCW235)*.

### Implementation of parkrun practice initiative

Practice staff were asked how they delivered the initiative, with EOs asked how they perceived that it was being delivered. Table 3 represents the delivery of the initiative to patients and staff (as reported by practice staff and as perceived by EOs).

**Table 3:** Activities adopted by parkrun practices to promote the initiative (separated by patients and carers and practice staff) compared against event organisers perceptions of adopted activities

	Practice Staff (*n* = 416)	Event Organisers (*n* = 207)
**Activities Adopted to Promote Initiative to Patients and Carers**		
**Regularly speaking to patient +/- carers about parkrun and the benefits of participating**	**165 (39.7%)**	**100 (48.3%)**
Presence of a parkrun noticeboard in the practice	162 (38.9%)	42 (20.3%)
Regularly sharing parkrun flyers (hard copy or digital)	142 (34.1%)	51 (24.6%)
Hosting information about parkrun on the practice website	117 (28.1%)	12 (5.8%)
Encouraging other initiatives that link with parkrun, like 5k Your Way	105 (25.2%)	28 (13.5%)
Highlighting parkrun in practice newsletters	87 (20.9%)	12 (5.8%)
Organising a day for staff and patients to attend a local parkrun together	83 (20.0%)	30 (14.5%)
Delivering a parkrun practice slideshow on practice TV screens	78 (18.8%)	19 (9.2%)
Using text-based messaging software to suggest parkrun to patients	67 (16.1%)	1 (0.5%)
**Delivering a presentation to patients/carers about parkrun and its benefits**	**16 (3.8%)**	**19 (9.2%)**
**Sharing parkrun and stories on social media platforms**	**8 (1.9%)**	**13 (6.3%)**
Other	151 (36.3%)	34 (16.4%)
Not known	17 (4.1%)	58 (28.0%)
**Activities Adopted to Promote Initiative to Practice Staff**		
Staff regularly attending parkrun	312 (75.0%)	146 (70.5%)
Staff accompanying colleagues to parkrun events	274 (65.9%)	47 (22.7%)
Practice-wide presentation on parkrun	193 (46.4%)	24 (11.6%)
Presence of a parkrun staff noticeboard	96 (23.1%)	24 (11.6%)
Staff hosting a volunteer take-over of the parkrun event	78 (18.8%)	38 (18.4%)
Sharing inspiration stories on their networks and wider	60 (14.4%)	7 (3.4%)
Sharing of case studies or practices that have linked with parkrun	38 (9.1%)	5 (2.4%)
Staff wellbeing challenges that reward parkrun participation	20 (4.8%)	3 (1.45%)
Other	49 (11.8%)	10 (4.8%)
Not known	17 (4.1%)	43 (20.8%)

**Fig. 1:**
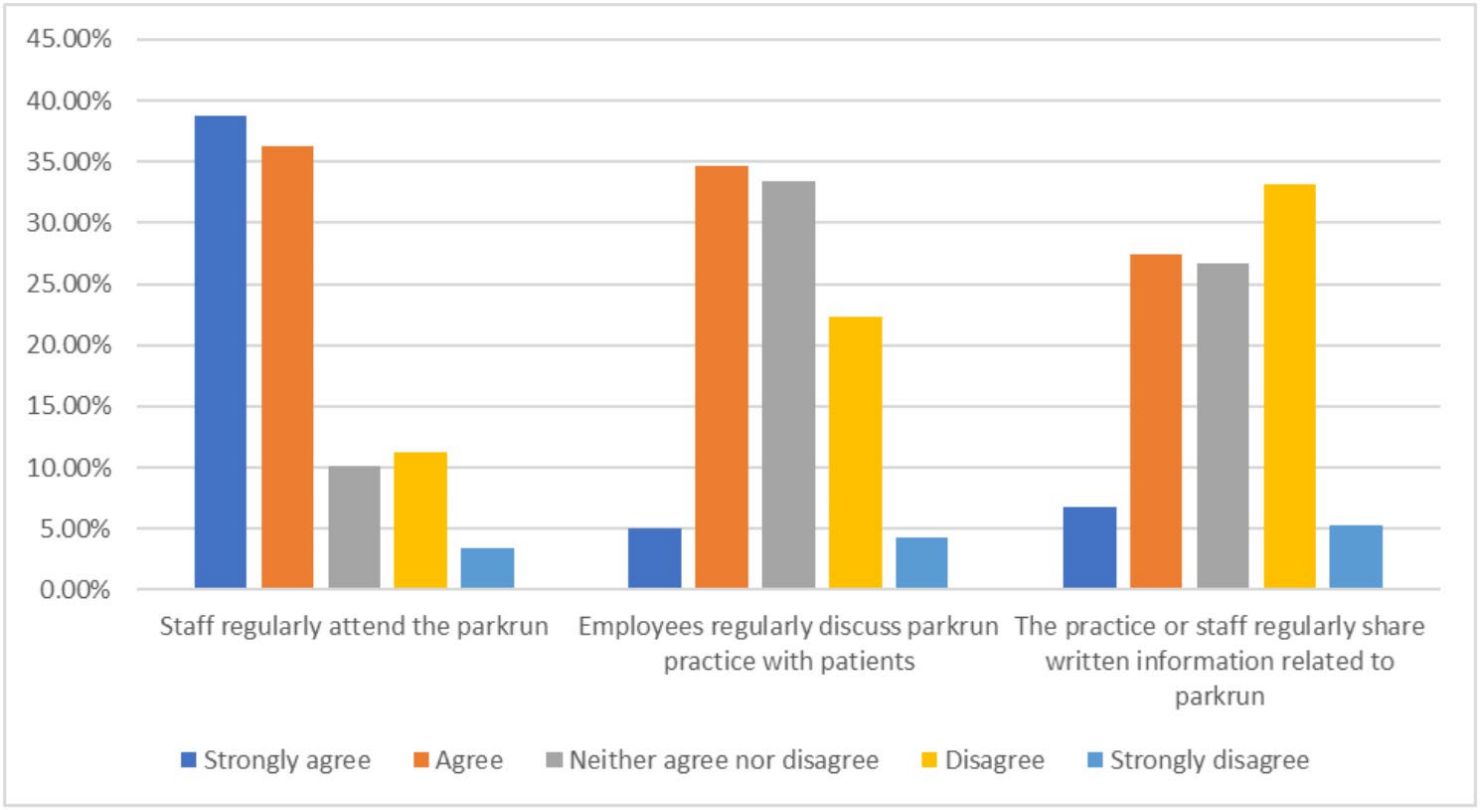
implementation of key elements of the parkrun practice initiative, as perceived by healthcare workers (represented via a Likert scale, number of respondents = 416)

Figure 1 shows the implementation of key elements of the *parkrun* practice initiative, as perceived by practice staff via a Likert scale.

### Perceived benefits of parkrun practice initiative

**Fig. 2:**
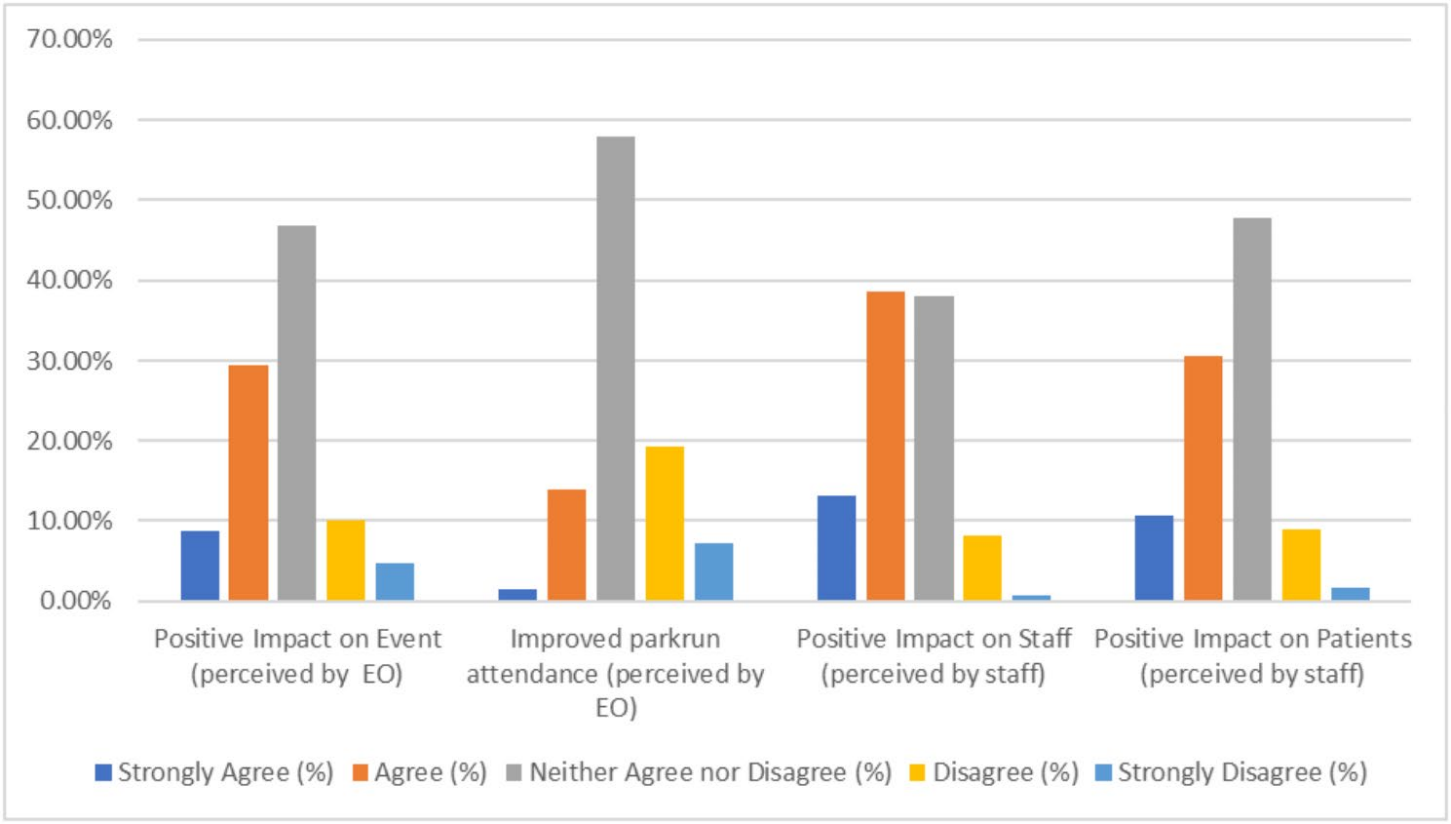
Likert scale assessment of the perceived impact of the parkrun Practice Initiative. This related to perceived impact by event organisers (*n* = 207) on overall event impact and attendance, and perceived impact by practice staff (*n* = 416) on colleagues and patients

Figure 2 represents the perceived benefits (as judged by EOs) of the *parkrun* practice initiative on attendance and the event generally, alongside the perceived benefits (as judged by practice staff) of the *parkrun* practice initiative on patient and staff wellbeing.

Practice staff that responded highlighted four main benefits of the *parkrun* practice initiative (Table 4). These were: (1) improvements to staff health and wellbeing (25.4%, *n* = 107); (2) improvements in patient health and wellbeing (22.8%, *n* = 95); (3) community engagement (13.9%, *n* = 58); and (4) improvements in staff morale (11.3%, *n* = 47).

**Table 4:** A summary of the qualitative content analysis for the survey assessing the perceived impact of parkrun practice initiative as experienced by practice staff

Theme	Codes included	Percentage of responses (*n* = 416) Raw value in brackets	Illustrative quotes:
Patient Benefits	Patient Health/Wellbeing	22.8% (95)	*“Patient health and wellbeing including community aspects and volunteering opportunities.” (HCW393)*
	Medication Avoidance	0.5% (2)	*“Deprescribing & demedicalisation.” (HCW6)*
Staff Benefits	Staff Health/Wellbeing	25.4% (107)	*“I think it’s been positive for staff and a shared bond between different members of the team that dont usually work together day to day.” (HCW28)*
	Staff Morale	11.3% (47)	*“Great opportunity to get team together in sport activity.” (HCW18)*
Parkrun/Community benefits	Community Engagement	13.9% (58)	*“Great to help those who buy into it creating community*,* purpose*,* belonging*,* conversation*,* as well as exercise.” (HCW9)* *“Increased community spirit and wellbeing.” (HCW33)*
	Staff Knowledge/Awareness	2.2% (9)	*“Increases awareness of park run.” (HCW46)*
	Improved Engagement	1.0% (4)	*“Increased talk about physical activity*,* increased participation in parkrun. It has also led to the staff sharing other activities*,* for example people have met for group cycles and walks and some of the ANPs are now talking of starting a badminton club.” (HCW163)*

Respondents highlighted the physical (improving physical function and fitness) and mental benefits (managing stress, improved mood) for staff alongside improved staff morale and social benefits from meeting together in a non-work environment.

*“I think it’s been positive for staff and a shared bond between different members of the team that don’t usually work together day to day” (HCW28)*.

*“Great opportunity to get team together in sport activity” (HCW18)*.

Responding practice staff also highlighted the role of the *parkrun* practice initiative in facilitating community engagement. Community engagement refers to both the health benefits of socialisation and feeling of belonging to a community, but also giving the practice a platform to have a visible presence within their community.

*“Great to help those who buy into it creating community*,* purpose*,* belonging*,* conversation*,* as well as exercise” (HCW9)*.

*“[…]We have an older demographic here and we believe building a stronger community and offering a chance for our older patients to socialises with other people will massively benefit the ones struggling with loneliness for example.” (HCW160)*.

### Barriers to implementation

Several barriers to delivery of the *parkrun* practice initiative were experienced by EOs. These are shown in detail in Table 5.

**Table 5:** A summary of the qualitative content analysis for the survey assessing the challenges experienced by event organisers (EOs) and practice staff in implementation of parkrun practice initiative

Theme	Codes included			Percentage of Responses (*n* = 439) Raw value in brackets	Illustrative quotes:		
Challenges Experiences by Event Organisers							
Resource Constraints (practice and event team):	Engagement amongst all practice staff			38.0% (167)	*“We are having a lot of problems engaging with our GP Practices*,* we have invited them to come to the NHS celebrations and tried to engage with them to ensure they have all the latest information*,* but we rarely get any response from them.” (EO70)*		
	Time/Workload			21.9% (96)	*“As a parkrun organiser I am weary of taking on more and more work. parkrun practice initiatives must not increase my workload to any significant extent.” (EO27)*		
	Initial GP enthusiasm with no follow up			7.3% (32)	*“Good initial interest rapidly falls away.” (EO170)*		
	Perceived staffing constraints within the practice			5.0% (22)	*“The practice incredibly busy and somewhat understaffed” (EO142)*		
Communication:	Ongoing communication			9.3 (41)	*“…there are no clear lines of communication between our event and the practices.” (EO277)*		
	Knowing who to contact			4.8% (21)	*“They keep changing their social prescribing personnel which is very frustrating” (EO143)*		
Lack of awareness/understanding regarding parkrun	Awareness			14.6% (64)	*“[lack of] awareness of the initiative within the practice.” (EO17)*		
	Lack of training/resources			12.5% (55)	*“Some more material and information to core teams and emails to help parkruns engage with practices would be helpful please. I think teams often forget about the parkrun practice initiative with everything else they need to think about.” (EO48)*		
Structure of the initiative	No formal referral process			3.9% (17)	*“Don’t really know what impact the GP practice is having on our parkrun. We don’t know who is coming specifically because of a recommendation by medical staff” (EO269)*		
	Organisational constraints			3.2% (14)	*“…so little support bearing in mind your asking volunteers to do the work.” (EO119)*		
External factors	COVID-19			4.1% (18)	*“It started off great but has been difficult to restart from covid.” (EO315)*		
	Geography			3.2% (14)	*“Our paired practice isn’t in the same town as our event.” (EO386)*		
**Challenges Experienced by Practice Staff**							
Resource Constraints (practice and event team)	Engagement			30.0% (125)	*“It is so hard to motivate the staff to join in. Many are very sedentary and overweight and have no desire to change.” (HCW24)*		
	Time/Workload			13.7% (57)	*“The challenges tend to come from within primary care*,* we are too busy to fully promote it within our own team.” (HCW34)* *“[difficulty] fitting the chat into busy consultations.” (HCW399)*		
Preconceptions and understanding:	Challenging preconceptions			6.3% (26)	*“Patients thinking they have to run when they can walk or volunteer.”(HCW107)*		
	Lack of (or lack of awareness of) resources/knowledge/training			5.5% (23)	*“Lack of promotional materials” (HCW081)*		
Structure of the initiative	Organisational constraints including no formal referral process			2.4% (10)	*“Difficult to capture data for patient participation to further promote parkrun.” (HCW225)* *“No formal referral system in place for patients.” (HCW23)*		
External factors	Geography			9.6% (40)	*“Main issue is not having a park run in our local catchment area*,* having to get public transport/ drive there would be a big deterrent to our patients.” (HCW258)*		
	COVID-19			5.8% (24)	*“Keeping up initiative and participation*,* initially was popular but dropped off over the pan demic.” (HCW102)*		
	Cancellations			2.2% (9)	*“The local Parkrun is often cancelled when it gets wet/muddy. Some of practice have helped with improving the course but it still gets cancelled often.” (HCW127)*		

The three most frequently cited challenges were: (1) a lack of time to aid delivery of the initiative (38.0%, *n* = 167); (2) a lack of engagement from local GP practices (21.9%, *n* = 96); and (3) a lack of awareness of the initiative amongst practice staff (14.6%, *n* = 64). Other barriers include a lack of resources/training, lack of a formal referral process and the impact of the COVID-19 pandemic.

Practice staff (Table 5) cited similar barriers, including: (1) a lack of engagement from colleagues (30.0%, *n* = 125), and (2) a lack of time to deliver the initiative (13.7%, *n* = 57).

A lack of engagement cited by practice staff was related to the barriers of (1) persuading practice managers and GPs to link the practice to a *parkrun*; (2) convincing staff of the benefits both for themselves and those they care for; and (3) the ongoing ‘buy-in’/enthusiasm to ensure longer term successful delivery.

*“It is so hard to motivate the staff to join in. Many are very sedentary and overweight and have no desire to change.” (HCW24)*.

Practice staff also cited geographical limitations (9.6%, n = 40) and the COVID-19 pandemic (5.8%, n = 20). Many practice staff described the linked *parkrun* being out of the practice catchment or staff not living near their practice (and therefore *parkrun*), with distance acting as a barrier.

*“[staff] often have to commute significant distance to work & hence parkrun*,* our practice location not ideal as not that close to parkrun” (HCW303)*.

Finally, respondents cited the profound impact of the COVID-19 pandemic on both personal and working lives, with an ongoing impact on workload. This has led a loss of momentum and prioritisation of the *parkrun* practice initiative:

*“[Challenge is] Covid!! Still in process of getting things back up and running since we lost that momentum in 2020 (and focus had to turn to purely clinical work)” (HCW367)*.

## Discussion

### Summary

This study provides an overview of the *parkrun* practice initiative in the UK, particularly related to motivations for registering as a *parkrun* practice, means of delivery, perceived benefits and barriers to delivery. Overall, *parkrun* practices felt the initiative positively impacted staff and patient health and wellbeing, whilst also highlighting improved staff morale and engagement with the local community.

47% (207/439) of EOs that responded reported their *parkrun* was paired with a GP practice. Despite guidance asking practices to contact the event team and get their consent prior to signing up, in reality this does not always take place and GP practices can sign up to the initiative without first contacting the local *parkrun* event team. Therefore, EOs may be unaware that their event has been linked to a practice through this initiative. A lack of clear communication between EOs and practices may also exist in established connections. Although, nearly 40% of EOs said that the initiative had positively impacted their event, this may have been greater if there had been better awareness of the activities being undertaken by linked practices (over 20% of *parkrun* events were not aware of what activities the practice had undertaken).

Two major challenges to implementation were noted across both groups: a lack of time, and a lack of engagement. EOs highlighted a lack of engagement by primary care staff in general, whilst practice staff highlighted a lack of engagement (initial and ongoing) by colleagues in the initiative. Practice staff also highlighted the impact of distance to the nearest *parkrun* (both personally and as a patient representative) and the disruptive effect of the COVID-19 pandemic (with subsequent increased workload) on healthcare delivery as barriers to implementation.

### Strengths and limitations

Although qualitative analysis of free text responses in this survey helped generate additional insight, particularly related to barriers and perceived benefits of the initiative, these were relatively superficial and a more in-depth understanding through semi-structured qualitative interviews or focus groups would have been able to expand upon the findings of this study in greater depth.

Although the response rate in both groups was relatively high (55% of all *parkrun* events and 22.5% of all *parkrun* practices), responder bias may have led to the inclusion of practices and EOs who were more committed to the initiative. Given the unprecedented pressures on healthcare professionals (especially at the time of the survey distribution), response rates and/or responder bias may have been amplified in these results. This may explain why, despite the involvement of incentives for participation, the response rate of practices was lower than the 2019 analysis (22.5% v 39.2%) [21].

### Comparisons to existing literature

Our findings highlight engagement and time constraints/workload as the main barriers to the implementation of the *parkrun* practice initiative. Lack of time has repeatedly been highlighted as a major challenge to PA promotion in primary care [17, 18]. The COVID-19 pandemic exacerbated this lack of time, leading to a crisis within primary care, facilitating rapid change: remote working, less face-to-face delivery, increased workload (vaccination roll out delivery, for example), and a worsening of the pre-existing staffing shortage [25]. Responders also directly highlighted the impact of COVID-19, with the pause in *parkrun* events breaking routine and the change in work patterns leading to a subsequent fall in engagement. The impact of the COVID-19 pandemic may also have affected sign-up to the *parkrun* practice initiative. The initiative started in 2018, with an initial larger number of earlier adopters (39.9% pre-April 2020, see Table 1), and subsequent recruitment was slower (aligns with the COVID-19 pandemic).

The *parkrun* practice initiative provides an opportunity for PA promotion, but there is potential that the lack of time and high workload (impacted by the pandemic) leave little capacity for healthcare workers to engage in delivering the initiative. Recent work evaluating another national initiative (RCGP Active Practice Charter) to promote PA in primary care settings [22] identified time, engagement, and costs as the main barriers to implementation– suggesting these factors as important considerations to address when refining current initiatives or developing new ones.

Practices reported undertaking a broad range of activities (as recommended within the ‘Toolkit’) to deliver the initiative (Table 3). However, the percentage of practices undertaking recommended activities to implement the initiative was fewer in our findings than those reported by Fleming and colleagues in 2020 [21]. This is particularly noticeable with reference to: (1) encouraging patients to take part in *parkrun* in consultation (40% v. 79%); sharing of flyers (34% v. 57%); (2) practice website pages (28% v. 40%); and (3) displaying information regarding *parkrun* on television screens in practice (19% v. 35%). As the first to sign up, the early adopters of the initiative evaluated in 2020 may have been more motivated, and this may explain some of the differences. However, given these changes, our findings provide insight into implementation in the current primary care context and highlight a possibility to reform the *parkrun* practice offering. The previous evaluation [21] did identify the two major barriers to implementation: (1) a lack of time; and (2) a lack of interest and enthusiasm by practice staff. Fleming and colleagues highlighted a need to determine ways to engage the wider practice team and engage with practices not familiar with *parkrun* or its benefits.

This study shows the wide variety of ways in which the *parkrun* practice initiative is implemented between practices. This flexibility is in-keeping with the Toolkit [19] and allows for inter-practice variations in needs, priorities and working-styles. Despite this, the key means of delivery were attendance at *parkrun*, discussion with patients/carers and colleagues, and sharing of information across a variety of media (e.g., social media, SMS, webpages). There appears to be a discrepancy between what practices report as being done and what EOs perceive as being done (Table 3). Except for “regularly speaking to patient +/- carer”, “delivering a presentation to patients” and “sharing parkrun and stories on social media platforms”, practice staff report implementing activities to promote *parkrun* much more frequently that EOs perceive it to be happening. This ‘perception gap’ may be explained by the lack of engagement of practice staff and communication failures which are cited as barriers to the initiative delivery by EOs, with steps to address these barriers providing potential for improving the initiative.

Almost 40% of EOs (Fig. 2) regarded the initiative as having a positive influence on their event, but a much smaller number (15%) identified a positive impact on attendance. Given the complexity of decision making, it is likely that a number of influences affect an individual’s decision to attend *parkrun*, making it very hard to ascertain the exact impact of the initiative on attendance (or PA more generally) [26]. At present, there is also no way of measuring referrals to *parkrun*, and so perceived impact on attendance is observational. Encouraging practice staff to code referrals/discussion may go some-way to addressing this. It is in this context of complexity that the International Society of Physical Activity for Health (ISPAH) highlight a need for a systems-based approach to PA [27], of which healthcare system engagement is an important part. Initiatives, like the *parkrun* practice initiative, are therefore important.

A 2019 evaluation (published in 2022) also explored EOs perceptions of the *parkrun* practice initiative [28]. In this study, EOs that had engaged in the initiative reported being motivated by wanting to positively impact the health and wellbeing of their community. In seeking to address the main barriers experienced by EOs in delivering the initiative (making initial contact with practices, lack of time and lack of clarity around responsibilities), Fleming and colleagues identified two key areas needing to be addressed: (1) establishment of clear communication pathways, and (2) developing support systems to minimise resource implications for EOs and practices to delivery of the initiative. Our study found that engagement of practice staff remained the biggest challenge. Further qualitative work, utilising behaviour change principles, would allow exploration of these barriers in more detail.

### Implications for research and practice

This work identified that the *parkrun* practice initiative is well received, but significant barriers to its successful delivery exist. These barriers align with those identified in evaluations of other initiatives to promote PA in primary care [22]. Future initiatives therefore need to address these, including: (1) clear and ongoing communication to ensure widespread engagement and adoption; (2) ease of implementation, minimising time and resource demands on practice staff and volunteers alike; and (3) adequate resource allocation to facilitate implementation (including financial, educational support, personnel). The National Health Service’s Long-Term Plan committed all primary care networks (PCNs) in England and Wales to provide a proactive social prescribing service [29]. Given the organisational intention of *parkrun* to address social isolation and loneliness, it presents an opportunity for social prescribing, and the *parkrun* practice initiative should seek to evolve to ensure social prescriber in PCNs have a central role in delivery. This may in turn minimise time demands on practice staff and volunteers and, if specifically addressed, help facilitate improved communication and engagement. The need to address these challenges moving forward, is particularly important given the value placed on prevention and community health within the recent report on the National Health Service by Lord Darzi [30].

With a further accumulation of evidence to support *parkrun* practices, including research to explore patient perspectives of the initiative, a targeted campaign could be adopted to support future expansion (and re-invigoration) of the initiative. With the inclusion of an educational element, this may address several identified barriers (including lack of engagement and lack of knowledge) but will benefit from an evidence-based co-production approach. Future iterations should be planned in accordance with a systems theory, with the soft-systems methodology (as an example) allowing for an individual and stakeholder driven approach [31].

Given the declining morale within the NHS workforce and the current ‘crisis’ in general practice [32, 33], the perceived benefits of the *parkrun* practice initiative on staff wellbeing and morale is promising. Alongside further research exploring this and patient outcomes, work is required to explore the psychological and behavioural mechanisms (of practice staff and EOs) influencing implementation. Finally, given the evidence that ‘active doctors make active patients’ [34–36], future initiatives should look to target (at least in part) healthcare professionals, and how to best utilise the potential of primary care staff as PA ‘advocates’.

## Electronic supplementary material

Below is the link to the electronic supplementary material.

Supplementary Material 1

## Data Availability

The dataset underlying this article is not publicly available as the study participants did not give consent for their data to be shared publicly. However, the anonymised data are available from the corresponding author upon reasonable request.

## Abbreviations

PA: Physical activity
EO: Event organiser
RCGP: Royal college of general practitioners
HCW: Healthcare worker
NHS: National health service
