# Supplementary material for: Motivations, delivery, perceived benefits, and barriers to delivery of the parkrun practice initiative in general practices across the UK: a national cross-sectional online survey of healthcare professionals and event organisers
Source: BMC Prim Care. 2025 Apr 29;26:137. doi: 10.1186/s12875-025-02827-9 (PMC12038959; doi:10.1186/s12875-025-02827-9)
Supplement: Supplementary file 1 — Supplementary Material 1 [file 12875_2025_2827_MOESM1_ESM.docx]

**Supplementary File 1: Survey**

Are you a primary-care practitioner or event organiser?

Primary-care practitioner or administration(Stem 1)

Event organiser (Stem 2)

**Stem One**

1.What is your current role?

| GP  Nurse  Practice Manager  Administration team  Doctor in training  Physiotherapist  Other [FREE TEXT] |
| --- |

2.How long have you been a parkrun practice?

| <1 year  1-2 years  2-3 years  4 or more years |
| --- |
|  |

3.Were you a parkrun participant prior to joining the initiative?

| Yes  No  Can’t remember |
| --- |

4.How is your practice list size?

| <4000  4000-7999  8000-11999  12000-15999  16000-20000  >20000 |
| --- |

5.How many parkruns are linked to the practice?

| 1  2  3  4 or more |
| --- |

7.What motivated the practice to register as a parkrun *practice*? *[FREE TEXT]*

8.Regarding making links with the local parkrun, how did this happen?

| Parkrun event made first contact |
| --- |
| Practice made first contact  Other [FREE TEXT] |

9.For the following statements please indicate your level of agreement: .

1. The parkrun practice initiative has led to improved patient health outcomes.

| Strongly agree |
| --- |
| Agree |
| Neither agree nor disagree |
| Disagree |
| Strongly disagree |

1. The parkrun practice initiative has improved staff health and wellbeing.

| Strongly agree |
| --- |
| Agree |
| Neither agree nor disagree |
| Disagree |
| Strongly disagree |

10.What activities have you done to enthuse and engage practice staff about parkrun? (select one or more)

| Practice-wide presentation on parkrun |
| --- |
| Share case studies of practices that have linked with parkrun |
| Accompany colleagues to parkrun events |
| Have a parkrun staff noticeboard |
| Organize staff wellbeing challenges that reward parkrun participation  Host a volunteer take over of a parkrun event  Share inspirational stories on their networks and wider  Other [FREE TEXT] |

11.What activities do you do to engage patients/carers about parkrun? (select one or more)

| Use text-based messaging software to suggest parkrun to patients |
| --- |
| Have a parkrun noticeboard in the practice |
| Deliver parkrun practice slideshow on practice TV screens |
| Deliver a presentation to patients/carers about parkrun and its benefits |
| Host information about parkrun on your practice website  Highlight parkrun in practice newsletters  Share parkrun and stories on social media platforms  Organise a day for staff and patients to attend a local parkrun together  Encourage other initiatives that link with parkrun, like 5k Your Way  Other [FREE TEXT] |

12 .For the following statements please indicate your level of agreement: .

1. A member of our practice regularly attends the local parkrun.

| Strongly agree |
| --- |
| Agree |
| Neither agree nor disagree |
| Disagree |
| Strongly disagree |

1. Practice employees regularly discuss parkrun with patients and carers.

| Strongly agree |
| --- |
| Agree |
| Neither agree nor disagree |
| Disagree |
| Strongly disagree   \| 1. We regularly share flyers, newsletters or social media posts promoting parkrun   Strongly agree \| \| --- \| \| Agree \| \| Neither agree nor disagree \| \| Disagree \| \| Strongly disagree \| |

13. Have you experienced any particular challenges in the delivery of the parkrun practice initiative? [FREE TEXT]

14.If willing, could you expand on any perceived positive impacts of the initiative? [FREE TEXT]

15.If willing, please provide the name of the practice you represent? All findings will remain anonymous but this will help us with future analysis. [FREE TEXT]

**Stem Two**

1 .Is the parkrun event you organised paired with a GP practice?

| Yes Question 2  No Question 8 |
| --- |

**Stem 2.1**

2.If so, how many GP practices are linked to your parkrun?

| 1  2  3  4 or more |
| --- |

3.Regarding making links with the local GP practice, how did this happen?

| Parkrun event made first contact |
| --- |
| Practice made first contact  Other |

4.For the following statements please indicate your level of agreement: .

1. The parkrun practice initiative has had a positive impact on the parkrun event.

| Strongly agree |
| --- |
| Agree |
| Neither agree nor disagree |
| Disagree |
| Strongly disagree |

1. The parkrun practice initiative has improved attendance at the parkrun event.

| Strongly agree |
| --- |
| Agree |
| Neither agree nor disagree |
| Disagree |
| Strongly disagree |
|  |

5.Are you aware of your linked GP practice engaging staff about parkrun in any of the following (select on or more)

Staff attending parkrun

Practice-wide presentation on parkrun

Sharing of case studies of practices that have linked with parkrun

Staff accompanying colleagues to parkrun events

Presence of a parkrun staff noticeboard in the GP practice

Staff wellbeing challenges that reward parkrun participation

Staff hosting a volunteer take over of the parkrun event

Sharing of inspirational stories on their networks and wider

Other [FREE TEXT]

6.Are you aware of the linked GP practice engaging patients/carers about parkrun in any of the following (select one or more)

Speaking to patients/carers about parkrun and the benefits of participating

Sharing parkrun flyers (hard copy or digital)

Using text-based messaging software to suggest parkrun to patients

Presence of a parkrun noticeboard in the practice

Delivering a parkrun practice slideshow on practice TV screens

Delivering a presentation to patients/carers about parkrun and its benefits

Hosting information about parkrun on the practice website

Highlighting parkrun in practice newsletters

Sharing parkrun and stories on social media platforms

Organising a day for staff and patients to attend a local parkrun together

Encouraging other initiatives that link with parkrun, like 5k Your Way

Other [FREE TEXT]

7.Are there any particular challenges to engaging and partnering with GP practices? [FREE TEXT]

**Stem 2.2**

8.Have you tried to partner with a GP practice?

Yes

No

9.If yes, what barriers or challenges have there been to partnering?

10. If no, is there a reason why not?

**Final Page**

Thank you for your participation in this survey assessing the parkrun practice initiative.

Your comments will help us as we look to improve the initiative.

If you have any further comments or queries please do not hesitate to get in contact with Dr Callum Leese via email at [cleese001@dundee.ac.uk](mailto:cleese001@dundee.ac.uk)

If you wish to be entered into our prize draw for Brookes running shoes, Shok's headphones and parkrun T-shirts then please leave your email below. The winners will be selected at random, with all email address deleted after the prizes have been allocated. Good luck!

*[FREE TEXT]*
